# Genome sequencing of *Enterococcus faecalis* strain 13, an oral strain isolated from C57BL/6 mice

**DOI:** 10.1128/mra.00185-24

**Published:** 2024-09-24

**Authors:** Roberto Vazquez-Munoz, Jordan T. Russell, Angela Thompson, Anna Dongari-Bagtzoglou

**Affiliations:** 1 Department of General Dentistry, University of Connecticut Health Center, Farmington, Connecticut, USA; 2 Pfizer, Groton, Connecticut, USA; Indiana University Bloomington, Bloomington, Indiana, USA

**Keywords:** *Enterococcus*, whole genome sequence, oral candidiasis, oral microbiome

## Abstract

*Enterococcus faecalis* strain 13 (Ef#13) is a tongue isolate from a C57BL/6 mouse with experimental oropharyngeal candidiasis. Short-read sequencing revealed a genome of 2,767,598 bp with a GC content of 38.4%, and 2,649 genes were predicted.

## ANNOUNCEMENT

Ef#13, isolated from the tongue of a C57BL/6 female mouse (Jackson Laboratories, USA; UCONN Health IACUC protocol #101640–0720) with 5-fluorouracil-associated oropharyngeal candidiasis, was identified as *E. faecalis via* PCR species-specific primers, detailed in Ref. (1). This strain expresses the metalloproteinase gelatinase E, degrades E-cadherin *in vitro*, and synergizes with *Candida albicans* to compromise the epithelial barrier integrity, facilitating fungal invasion (1). Ef#13 planktonic growth is inhibited by the antifungal *Lactobacillus johnsonii* strain MT4 in cocultures (2–4).

### DNA purification

Ef#13 was isolated by plating tongue homogenates in De Man–Rogosa–Sharpe (MRS) agar and incubating anaerobically for 24 h at 37°C. Bacterial DNA was extracted from frozen cultures grown in BHI broth (overnight, anaerobically, 37°C), using a pre-lysis step (4) and the DNeasy Blood and Tissue purification kit (Qiagen, Germany), following the manufacturer’s instructions.

### Sequencing

Genomic DNA (gDNA) was quantified (dsDNA High-Sensitivity Assay for Qubit 3.0, Life Technologies, USA) and analyzed for fragmentation (Genomic DNA assay, Agilent 4200 TapeStation, Agilent Technologies, USA). 1 ng of gDNA was normalized to 0.2 ng/µL for whole genome shotgun library preparation (Nextera XT Library Preparation kit, Illumina, USA), following the manufacturer’s instructions. Libraries were validated for length (450 bp average length; average insert size 315 bp). The High-Sensitivity D5000 ScreenTape assay (Agilent Technologies) was used for Adapter dimer removal. Libraries were quantified, normalized (dsDNA High-Sensitivity Assay for Qubit 3.0), and prepared for sequencing by denaturing and diluting the libraries (Illumina protocol). The sample was run on the Illumina NovaSeq SP 300 cycle sequencing kit v1.5. Target read depth was achieved with paired-end 300-bp reads. Sequencing reads were filtered based on Illumina Basecalling software algorithms. SprayNPray v1.0 was used for taxonomic profiling of the contigs and filtering and removing contaminants (5). The forward and reverse FASTQ sequences were analyzed for quality control (FASTQC, scores > Q30), and no further QC was performed. PhiX reads and Nextera adapter sequences were filtered from both paired-end FASTQ files using USEARCH v10.0.240 (6) and Trimmimatic v0.39 (7), respectively.

### Assembly and annotation

2,080,008 read pairs were obtained. Reads from both paired-end FASTQ files were analyzed with BBDuk (BBTools package) (8) to remove any contamination and assembled *de novo* using the Genome Assembly Algorithm SPAdes v.3.15.2 (9) (default parameters, Unicycler v0.4.8). The final assembly consisted of 66 contigs (N50 = 117.608 kb), with a length of 2,767,598 bp (GC content 38.4%) in a single genome. The coverage averaged 556×. The annotation was added by the NCBI Prokaryotic Genome Annotation Pipeline rev_6.5 (10). Moreover, 2,649 genes were predicted: 2,558 protein-coding, 56 miscellaneous RNA, and 35 pseudogenes. The genome contains one CRISPR array.

### Classification

Average nucleotide identity (ANI) analysis in the NCBI RefSeq database against 503 *E. faecalis* genomes confirms Ef#13 identity (99.93% ANI). Its complete genome was compared with all publically available *E. faecalis* strains. *E. faecalis* strain FDAARGOS_611, associated with food poisoning (11), is related to Ef#13.

## Data Availability

BioProject PRJNA963055 contains Ef#13 BioSample SAMN34433758, raw SRA (SRR24459547), and SRA decontaminated (final) (SRR24349409). The assembled genome is here.
